# Supplementary material for: A qualitative study on knowledge, perception, and practice related to non-communicable diseases in relation to happiness among rural and urban residents in Bhutan
Source: PLoS One. 2020 Jun 29;15(6):e0234257. doi: 10.1371/journal.pone.0234257 (PMC7323992; doi:10.1371/journal.pone.0234257)
Supplement: S1 File — (DOCX) [file pone.0234257.s001.docx]

**Supplements**

**Appendix 1-1: Explanation sheet**

**Appendix 1-2: Informed consent sheet**

**Appendix 2-1: Interview flow**

**Appendix 2-2: Interview Objective**

**Appendix 2-3: Interview questionnaire**

**Appendix 3: Observation sheet**

**Appendix 4:Diet photography sheet**

**Appendix 5: Activity monitoring sheet**

**Appendix 6: Daily Living Activity Record example**

Appendix 1-1: Explanation sheet

**Information　Sheet (Informed Consent)**

**Introduction**

I’m Hiromi Segawa from Japan. I’m public health nurse and Master of Public health candidate. Khesar Gyalpo University of Medical Sciences of Bhutan and Kyoto University in Japan are collaborating and trying to create sustainable society and well-being society for all. We would like to conduct a research and get your corporation. This research was approved by Kyoto University · Medical Ethics Committee and Research Ethics Board of Health, Ministry of Health and also obtained permission from Khesar Gyalpo University of Medical Science of Bhutan and Kyoto University Research Institute Head.

**Purpose of the research**

To explore how the socio-economic development had affected the health and happiness of the people of Phuntsholing town and Rangaytung community, Bhutan.

**Type of research intervention**

- Interview will take place in the place (home etc.) desired by you. After consenting for the

research study the interview and recording of the interview with IC recorder will take place.

- After the interview, the monitoring of your body condition (height, weight, body fat, blood pressure, abdominal circumference) and 7 days daily activity by parameter, calorism.)
- After due permission from the participant the researcher will staying with them for 1day to observe about life style (for example taking picture of your dish, daily activity ,cooking style, working style etc)

**Participant selection**

We are inviting all above 18years to below 60years old. And we pick up randomly by house of survey records or introduced by friends.

**Voluntary participation**

Your participation in this research is entirely voluntary. It is your choice whether to participate or not. Whether you choose to participate or not, all the services you receive at this clinic will continue and nothing will change. If you choose not to participate in this research project, you will offered the treatment that is routinely offered in this clinic/hospital for disease Z, and we will tell you more about it later. Just let us know if you want to discontinue to participate at any point of time, that is fine.

**Duration**

The research takes place over 7 days. ( / /2017)

☆You need to spend time around１hour for interview and accompany stay 1 day only, but after that you can stay as usual for 7 days with calorism. The survey period will be decided between May and August 2017, in consultation with you for your convenient schedule.

**Risk**

There is no risk as such by participating in this research, if you have lets us know, we will try to help you. Also you have right to discontinue.

**Benefits**

You can get some knowledge and get experience of health monitoring and also you will know about your health status.

**Reimbursements**

We will give you Nu. 200 as a compensation for your time. This amount will be given, when we receive back the parameter Calorism) after 7days. Other than this, you will not be given any other money or gifts to take part in this research. This research is funded by the Ministry of Education, Culture, Sports, Science and Technology doctoral program educational reading program funded through the GSS program. We have no conflict in this research.

**Confidentiality**

The information that we collect from this research project will be kept confidential. Any information about you will have a number on it instead of your name; only the researchers will have access to it and all the information will be kept under lock and key. The survey data will be kept for 10 years after the report / dissertation writing is finished, and as soon as these are completed, the acquired data will be erased from the personal computer.

**Sharing the result**

Confidential information will not be shared. After research meetings, we will publish the results and will be share to relevant authorities to promote health and prevent diseases in the country.

**Right to refuse or withdraw**

You do not have to take part in this research if you do not wish to. You may also withdraw from the research at any time you choose, it is your choice and all of your rights will be respected.

**Who to Contact**

If you have any questions about the research study, you are welcome to contact following individuals:

Hiromi Segawa, PHN, CM, RN, MPH candidate

Department of Global Health and Socio-epidemiology

Kyoto University School of Public Health

Yoshida Konoe-cho, Sakyo-ku, Kyoto 606-8501

Office TEL: +81-75-753-4350

Email: segawa.hiromi.57r@st.kyoto-u.ac.jp

Kunzang Dorji, Sr.Lecturer (Public Health)

E-mail: [kdorji@rihs.edu.bt](mailto:kdorji@rihs.edu.bt)

Khesar Gyalpo University of Medical Sciences of Bhutan, Thimphu

P.O.Box 298, Bhutan

PABX: +975-02-321212, 321210 (ext:120) Mobile No. +97517613695

This proposal has been reviewed and approved by Kyoto-University, which is a committee whose task it is to make sure that research participants are protected from harm.

If you wish to find about more about the institutional review board (IRB),

contact （ Mon - Fri ）8:30-17:00 JST

Office TEL：+81-75-753-4680

Office FAX：+81-75-753-4642

E-mail: [ethcom@kuhp.kyoto-u.ac.jp](mailto:ethcom@kuhp.kyoto-u.ac.jp)

This proposal has been reviewed and approved by royal government of Bhutan ministry of health research ethics board of health Thimphu: Bhutan, which is a committee whose task it is to make sure that research participants are protected from harm.

Office TEL：+975-2-322602,322351,328091,3228092,3228093

Office FAX：+975-2-324649

**Certification of consent**

**Appendix 1-2: Informed consent sheet**

I have read the information, or it has been read to me. I have had the opportunity to ask questions about it and any questions that I have asked have been answered to my satisfaction. I consent voluntarily to participate as a participant in this research.

**Print Name of Participant__________________**

**Signature of Participant ___________________**

**Date ___________________________　　　　　　　　　　　　　　　　　　　　　　　　　　　　　　　Day/month/year**

**If illiterate**

I have witnessed the accurate reading of the consent form to the potential participant, and the individual has had the opportunity to ask questions. I confirm that the individual has given consent freely.

**Print name of witness_____________________ AND Thumb print of participant**

**Signature of witness ______________________**

**Date ________________________**

**Day/month/year**

**Statement by the researcher/person taking consent:**

I have accurately read out the information sheet to the potential participant, and to the best of my ability made sure that the participant understands the above information.

I confirm that the participant was given an opportunity to ask questions about the study, and all the questions asked by the participant have been answered correctly and to the best of my ability. I confirm that the individual has not been coerced into giving consent, and the consent has been given freely and voluntarily.

**Print Name of Researcher****/person taking the consent________________________**

**Signature of Researcher /person taking the consent__________________________**

**Date ___________________________　　　　　　　　　　　　　　　　　 Day/month/year**

Appendix 2-1: Interview flow

**Interview flow**

＊**This interview is semi-structured interview. Interviewer is trying the following points, but the interviewer may change the question items and order according to the situation of participants.**

First we’ll collect the information from House of survey data (Questionnaire until No 25).

And we will visit each house; get informed consents from the participants who are eligible and willing to participate in the research. We will discuss and decide the day of interview.

After interview we will check their body weight, height, fat, blood pressure and abdominal circumference and start to accompany observation. And at the end of the day we will give them the calorism to keep with them for 7 days.

Dear

We will visit your house on / /

Time:

If you want to contact with us, please call

No .

Thank you for your corporation.

Hiromi Segawa

Appendix 2-2: Interview Objective

**INTERVIEW OBJECTIVE GUIDE FLOW**

| **Categories** | **Items** | **Probes** |
| --- | --- | --- |
| **Dietary habit and preferences** |  |  |
| Decision-making | Who usually prepares the food that you eat? |  |
|  | Do you sometime have a choice over what you eat? |  |
|  | Do you prefer making your own choice or letting other people choose what you eat? |  |
|  | How do you decide about the place to buy food? | [What influence your choice for the place to buy food?] |
| Variety | What kind of food do you usually take? [breakfast, Lunch, Dinner]   - Vegetables - Proteins - Fruits - rice - Snacks |  |
| Quantity | Do you often take breakfast, lunch, dinner? |  |
|  | Are you satisfied with the quantity of the food you eat every day? | - Yes - No [small? Or big quantity? Why?] |
|  | Are you satisfied with the quantity of the food you eat every day? | - Yes - No [small? Or big quantity? Why?] |
|  | Do you think you consume enough water on average per day? | - Yes - No [small? Or big quantity? Why?] |
| Substance use | Do you take alcohol? | If yes,  How often?  How much?  How old did you start?  Yes, No  How do you think about taking alcohol? |
|  | Do you take cigarette? | If yes,  How often?  How much?  Yes, No  How do you think about taking alcohol? |
|  | Do you take doma? | If yes,  How often?  How much?  Yes, No  How do you think about taking alcohol? |
| Socialization and food habit | Where do you usually take breakfast, lunch, dinner?[at home? Workplace; restaurant?] |  |
|  | With whom? [Alone, family members, friends or colleagues?] |  |
|  | Do you often go to restaurant? | - What kind of restaurant? - How often? - Why? With whom? |
|  | What do you think are the benefits of taking food? | - Nutritional benefits? - Socializing with family members? friends?   Etc. |
| Changing habit | Do you think that eating habit was changed from childhood? | How and why? |
| **Perceptions related to dietary habit** |  |  |
| Beliefs | Do you believe that some foods can be good for your health? |  |
|  | Do you believe that some foods can be bad for your health? |  |
|  | Do you believe that some foods should be eaten [or not] for religious reasons? |  |
|  | Do you trust the place you buy food? [safe?] |  |
| **Knowledge related to diet and nutrition** |  |  |
| Knowledge | Have you ever heard about “balanced diet? |  |
|  | How did you learn about it (balanced diet)? |  |
|  | In case you heard about it, have you ever try to adopt a balanced diet in your eating habit? | -Yes [why?]  - No [why?] |
|  | Do you know some foods that can be bad for your health? |  |
|  | Do you know some foods that can be good for your health? |  |
| Knowledge of substance | Do you know the alcohol’s risk? |  |
|  | Do you take the cigarette’s risk? |  |
|  | Do you take the doma’s risk? |  |
| **Condition of daily life and activity** |  |  |
| Exercise behavior | Do you exercise? | If yes,  - how often?  -Are you happy with this behavior?  -What are the reasons you exercise?  If no,  -why?  -Have you ever considered exercising? |
| Exercise behavior  Sleeping behavior | What do you think are the benefits of physical exercise? | How you learned about exercise? |
|  | How do you plan your physical activity? | Do you balance your physical activity with food intake behavior? |
|  | What time do you usually go to bed? |  |
| Sleeping behavior  Job related activities | What time do you usually wake up? |  |
|  | Are satisfied with current job? | - If yes, what makes you feel so?   If no, why? |
| Job related activities  Leisure time | Would you consider that your job is stressful? | - If yes, how? How do you cope with the stress? |
|  | How do you commute to your working place? | On foot? Routes conditions (mountains, valleys, etc.} |
|  | How long does it take you to reach your working place? |  |
|  | How do you spend time on your holiday? |  |
| **Health belief and Action** |  |  |
| Health belief | What do you do to keep your body healthy? | Any specific tradition for health? |
| History of consultation | Where do you or your family generally receive health care when you are not feeling well? | -hospital, clinic , health center  - Traditional hear |
|  | Do you have a particular condition for which you often to see a doctor? | [What kind of advices do you receive from care provider]  - self-treatment |
| Consultation behavior | Do you do regular health check-up? | If yes,  -What kind of health check-up do you get?  -Where do you get?  -How many times per year?  -What is the motivation? |
| **Health literacy and Knowledge** |  |  |
| literacy | Where do you generally obtain information on health in case of need?  Is it easy for you to understand health information [from TV, radio, Internet, etc.] | Satisfied with the source of info? Why?  Do you trust the information [from different sources]  What kind of health information would you want to receive?  From which channel [radio, TV; Internet, friends, health providers, etc.]  -Internet access? |
| Knowledge level | Have you heard about life style disease? |  |
|  | If Yes, do know some life style diseases? [could you kindly cite them] | Diabetes; Cancer; Stroke; Chronic respiratory diseases; etc. |
|  | What do think make people develop those life style diseases? | High blood pressure; overweight obesity, pressure, physical inactivity, tobacco smoking |
|  | Do you think these diseases can be prevented? |  |
|  | What do you think can make it hard for you to prevent these diseases? | - Traditional lifestyle difficult to give up? |
|  | If you can prevent your disease, do you want to prevent? |  |
| Body image | What do you think about obesity or low-weight? | Why? |
| **Value of Happiness** |  |  |
| Determination of happiness | What generally makes you happy? | How about money?  ( If possible mention about Income and note to house of survey sheet)  [Is health connected your happiness?] How? |
|  | What is the most important thing in your life? | If you have some message, could you teach me? |
| Lifestyle and happiness | Where do you a think a person with an incurable disease should receive care {family? Friends?etc….} | Do you happen to know someone with the same condition? |

**Appendix 2-3: Interview questionnaire**

QUESTIONNAIRE FORM for Attribute

2. Date of recording

1. Questionnaire No

3. Recorded by

4. Address Area

8. Family composition

Town / Rural

5. Age

6. Sex

7. House Income

9. Income/Year

10. Type of house

1. House 02. Part of house 03. Separate apartment

04 Shared apartment 05 Hut/Bago 06 others

05. Living together 06. Unknown

11. Marital status

1. single 02. Married 03. Widowed 04 Divorced/Separated

05. Living together 06. Unknown

12. Occupation

1. Business 02. Agriculture farmer 03.Livestock farmer

04. Harvest of forestry product 05. Farm-hand 06.House maid/baby sitter

07. Student 08. Monk/Nun/Religious figures 09.Goverment service

10. Armed force 11.Occupation:None 12.Occupation: others

13. Education

1. Pre-primary/ECCD/Daycare Grade01 02 03 04 05 06 07 08 09 10 11 12

13.Certificate 14. Diploma 15. Bachelor degree 16.Master degree 17.Non-formal education 18 Gomchen/monk/nun 19 No-education 20 others

14. Farming

1. Agriculture 02. Livestock

03.grew at least 3 varieties of vegetables in last season

1. Electric 02.gas stove 03.lerosene stove 04.Firewood 05.Biogas

06.Cooking source :others

15. Cooking fuel

16. Health seeking behavior (Copy from house of survey records)

1. Household members sick in the past 1 month
2. Treatment: nobody/self-treatment
3. Treatment: Health professionals/facilities
4. Treatment: Drungthos/Menpas/Indinenous
5. Treatment: VHWs
6. Treatment: Lam/Lopen/Tsip/Pow/Local healers
7. Treatment: Did nothing
8. No of house hold consume/intake iodized salt
9. No of persons currently take alcohol regularly
10. No of persons currently smoke Cigarettes/bidis /cigars .
11. No of persons currently chew Tabacco
12. No of persons currently eat Betel quid with tobacco.
13. No of persons currently use Snuffs by nose

17. Health care expenditure (Copy from house of survey records)

1. Prescription drugs
2. Non-Prescription drugs
3. Hospital cabin charge
4. Transportation
5. Dental
6. Rimdos/pujas/religious
7. Others

18. Latrine type used

1. Pit without slab and/or cover 02. Pit with slab and cover 03. VIDP 04. Pour flush
2. Compost toilet with cover-Eco-san 06. Latrine: Shared 07. Latrine: None

05. Living together 06. Unknown

19. Toilet design prevents

1. Faces contact to human/animals 02. Access to files 03.Surface/grand water contamination

20. Toilet used

1. Intended purpose-urination/defecation 02 .Free of feacal smears and kept clean
2. Adequate privacy for all users

21. Waste disposal

1. Public garbage collection 02. Burning 03.Composting 04.Open Pit 05. Others

22. Sanitation others

1. Proper drainage 02. Live stock with separate animal-shed 03. Live stock without separate animal shed 04. Exposure to indoor smoke

23. Hand-washing facilities

1. Bowl/container of water- with possibility of contamination 02. Bowl/container of water –with NO possibility of contamination 03.Runnnig water-eg.tap stand, tippy tap

24. Availability: piped water

1. Piped water with Tap stand 02.piped water without Tap stand 03. Piped water with Supply Line 04. Piped water without Supply line 05. No piped water supply

25. Availability :Piped water

1. Piped water with functional Tap stand 02. Piped water with non-functional Tap stand

03.Piped water with functional supply line 04. Piped water with non-functional Supply line

**<Example>**

**Introduction and Ice breaking**

Introducing each other.

We try to make interviewee to feel comfortable and relax. We start with talking about culture different Bhutan and Japan followed by interviewers experience while staying in Bhutan. Interviewer would like to explore on their back ground, lifestyle and actual feeling of individuals.

**Dietary habits and preference**

1. Who prepare for your dish usually?

Additional: sexual different, cast or economic states?

2. What time do you take food? What kind of food do you take?

0 1 2 3 4 5 6 7 8 9 10 11 12 13 14 15 16 17 18 19 20 21 22 23

Do you take something between breakfast and lunch or dinner? If yes, what kind of food?

3. What kind of food do you like or don’t you like?

Additional: Do you think your favorite foods are your soul or traditional food?

　　　　　Do you feel the differences between your childhood and present? If yes, How?

4. Do you know the amount and contents of your food and water?

Yes or No

Do you consider about that or not? Who is the decision maker?

How about rice, vegetable, cheese, oil, chili, meal, fat, salt, sugar, pure water, juice, snacks etc

5. Where do the food resources come from?

Do you consider about the place of product? Yes or No. If yes, why?

6. Do you take alcohol? cigarett? doma?

If yes, when did you start? How much do you take per week?

How do you feel about taking these things? And why?

7.With whom do you take food? Break fast, Lunch, Dinner?

How do you feel about taking food? And why?

In your life what role is the taking food?

8. Are you used to take food from outside restaurant?

If yes, how often?

How do you feel about taking food from restaurant? And why?

9.Have you heard about nutrition?

If yes, what is the resource?

Are you interested about nutrition? If yes, Why?

Usually do you try something for good balance nutrition? Why?

Do you have something you believe that is good or bad food for health? why?

**Condition of daily life and daily activities**

10. Your job is ~~~, how do you feel about your job?

Is it stressful or not?

If yes, What is the stressor? And how you can overcome the stress?

What is the role of job in your life?

How do you go to working place?

How many minutes do you walk per day?

11. Do you have some habit of exersice?

If yes, what kind of exercise?

How many times do you do per week and hours?

How do you feel the habit of exercise? Are you happy with the exercise?

12. How do you spend time on your holiday/ free time?

13. How many hours do you sleep per day? (Average)

14. Have you learn about exercise?

If yes, from where and when?

15.Have you consider about your daily activity?

**Health belief and Action**

**Health literacy and knowledge of lifestyle disease**

16. Do you have some habit for keeping your body healthy?

If yes, what kind of habit?

In your opinion, what factors are good for health? Or bad? Why?

Do you know some traditional action for good health? What?

17. If you have some questions about your health, what do you do first?

Why?

If you ask somebody, whom do you ask? Why?

18. Do you have any medical history?

Yes/No

If Yes, What kind of medical history do you have?

After you get your treatment, did you change your life style?

If yes, How did you change?

How do you feel the treatment of hospital?

How do you feel the treatment of traditional medicine?

19. When your family or you become sick, how do you do usually?

Why?

20. What do you think about obesity or low-weight? * Showing the pictures and talk.

Why?

21. Have you heard about life style disease?

If yes,

From where did you get the information?

If you have high blood pressure, low weight, obesity, what kinds of disease risk are high?

Have you heard about diabetes? If yes what kind of disease?

If you have diabetes, what kinds of disease risk are high?

22. Do you do regular health check-up?

If yes, what kind of health check-up do you get? Where do you get? How many times per year?

**Value of Happiness**

23. What factor determines your happiness?

How much important of health for your happiness? How about money?

( If possible mention about Income and note to house of survey sheet)

Due to prevent some diseases, If you have to change your traditional life style, how do you feel?

If you can prevent your disease, do you want to prevent?

Do you know somebody become incurable sickness? How do you feel their happiness? If you have any sickness that is incurable, what do you want in your life?

24. What is the most important things for your life?　If you have some message, could you teach me?

**Interview Closing**

Thank you so much for your corporation. This is the last question, If you have a chance to learn about health, do you want to learn?

If you want to tell something more, please let me know.

⇒to shift the body measurement

Abdominal circumstance: cm

BMI:

Height: cm

BP: /

Weight: kg

Body Fat: %

**Observation sheet**

**Appendix 3: Observation sheet**

Before staring research, we will explain participants to keep usual lifestyle.

Observation points

1. Environment(Home, working place, transportation, town, village, shopping etc)
2. Social factors that affect daily activities
3. The food environment such as suppliers, food stuffs, preparations for meals, water snacks, alcohol, cigarette, doma etc
4. Their emotion (what time are they feeling satisfy or not)
5. Their social capital
6. Time use
7. Health behaviors
8. Religious behaviors

＊If I have some rest question from In –Depth-Interview, I’ll try to observe the points.

We don’t have to fill the sheet and during observation researcher is able to write 8 points. Following form is just example.

For example, with whom do they take lunch, where and what time. How is participant’s mood? What kind of chatting do they do during lunch? How many minutes do they take for lunch. How do they go to lunch place? How is environment of lunch? etc

| Time | Contents | Memo |
| --- | --- | --- |
| 0 |  |  |
| 1 |  |  |
| 2 |  |  |
| 3 |  |  |
| 4 |  |  |
| 5 |  |  |
| 6 |  |  |
| 7 |  |  |
| 8 |  |  |
| 9 |  |  |
| 10 |  |  |
| 11 |  |  |
| 12 |  |  |
| 13 |  |  |
| 14 |  |  |
| 15 |  |  |
| 16 |  |  |
| 17 |  |  |
| 18 |  |  |
| 19 |  |  |
| 20 |  |  |
| 21 |  |  |
| 22 |  |  |
| 23 |  |  |

**Diet photography**

**Appendix 4: Diet photography sheet**

|  |  |  |  |  |  |  |  |  |  |  |  |  |  |  |  |  |  |  |  |
| --- | --- | --- | --- | --- | --- | --- | --- | --- | --- | --- | --- | --- | --- | --- | --- | --- | --- | --- | --- |
|  |  |  |  |  |  |  |  |  |  |  |  |  |  |  |  |  |  |  |  |
|  |  |  |  |  |  |  |  |  |  |  |  |  |  |  |  |  |  |  |  |
|  |  |  |  |  |  |  |  |  |  |  |  |  |  |  |  |  |  |  |  |
|  |  |  |  |  |  |  |  |  |  |  |  |  |  |  |  |  |  |  |  |
|  |  |  |  |  |  |  |  |  |  |  |  |  |  |  |  |  |  |  |  |
|  |  |  |  |  |  |  |  |  |  |  |  |  |  |  |  |  |  |  |  |
|  |  |  |  |  |  |  |  |  |  |  |  |  |  |  |  |  |  |  |  |
|  |  |  |  |  |  |  |  |  |  |  |  |  |  |  |  |  |  |  |  |
|  |  |  |  |  |  |  |  |  |  |  |  |  |  |  |  |  |  |  |  |
|  |  |  |  |  |  |  |  |  |  |  |  |  |  |  |  |  |  |  |  |
|  |  |  |  |  |  |  |  |  |  |  |  |  |  |  |  |  |  |  |  |
|  |  |  |  |  |  |  |  |  |  |  |  |  |  |  |  |  |  |  |  |
|  |  |  |  |  |  |  |  |  |  |  |  |  |  |  |  |  |  |  |  |
|  |  |  |  |  |  |  |  |  |  |  |  |  |  |  |  |  |  |  |  |
|  |  |  |  |  |  |  |  |  |  |  |  |  |  |  |  |  |  |  |  |
|  |  |  |  |  |  |  |  |  |  |  |  |  |  |  |  |  |  |  |  |
|  |  |  |  |  |  |  |  |  |  |  |  |  |  |  |  |  |  |  |  |
|  |  |  |  |  |  |  |  |  |  |  |  |  |  |  |  |  |  |  |  |
|  |  |  |  |  |  |  |  |  |  |  |  |  |  |  |  |  |  |  |  |
|  |  |  |  |  |  |  |  |  |  |  |  |  |  |  |  |  |  |  |  |

Date:

Time:

Survey Number:

Each square is 1cm×1cm

We put dishes on the like this paper, and take picture.

**Appendix 5: Activity monitoring sheet**

**Daily Living Activity Record**

**Survey number:**

| Time | / | / | / | / | / | / | / | / |
| --- | --- | --- | --- | --- | --- | --- | --- | --- |
| 0 |  |  |  |  |  |  |  |  |
| 1 |  |  |  |  |  |  |  |  |
| 2 |  |  |  |  |  |  |  |  |
| 3 |  |  |  |  |  |  |  |  |
| 4 |  |  |  |  |  |  |  |  |
| 5 |  |  |  |  |  |  |  |  |
| 6 |  |  |  |  |  |  |  |  |
| 7 |  |  |  |  |  |  |  |  |
| 8 |  |  |  |  |  |  |  |  |
| 9 |  |  |  |  |  |  |  |  |
| 10 |  |  |  |  |  |  |  |  |
| 11 |  |  |  |  |  |  |  |  |
| 12 |  |  |  |  |  |  |  |  |
| 13 |  |  |  |  |  |  |  |  |
| 14 |  |  |  |  |  |  |  |  |
| 15 |  |  |  |  |  |  |  |  |
| 16 |  |  |  |  |  |  |  |  |
| 17 |  |  |  |  |  |  |  |  |
| 18 |  |  |  |  |  |  |  |  |
| 19 |  |  |  |  |  |  |  |  |
| 20 |  |  |  |  |  |  |  |  |
| 21 |  |  |  |  |  |  |  |  |
| 22 |  |  |  |  |  |  |  |  |
| 23 |  |  |  |  |  |  |  |  |
| Burning fat |  |  |  |  |  |  |  |  |
| Consumption |  |  |  |  |  |  |  |  |
| Distance |  |  |  |  |  |  |  |  |
| Steps |  |  |  |  |  |  |  |  |
| Run |  |  |  |  |  |  |  |  |
| walk |  |  |  |  |  |  |  |  |
| daily |  |  |  |  |  |  |  |  |
| Sleeping |  |  |  |  |  |  |  |  |
|  |  |  |  |  |  |  |  |  |

**Appendix 5: Calorism**

**What is Calorism(high mechanism of parameter)?**

Calorism Expart is just a smart way to check your calories: this micro gadget will monitor. "Analysis graph" that you can see at a glance the results you gained. You can understand walking time, jogging time, distance, calorie expenditure, number of steps, running steps, fat burning amount. Automatically distinguish your "walking" and "running". A 24-hour graphical display that shows the amount of activity every hour, you can see at a glance the actions of the day.

You can clip the Calorism to your clothes, put it in your pocket or even attach it as a magnet. A mere 38g (including batteries!) in weight means this is very portable, and the simple four-button functionality also means it is easy to operate too. You can even display and check graphs of your health data from the last 24 hours and 2 weeks.

After through explanation about the use of Calorism, the participants will be given a Calorism machine to record their day to day physical activities. This measure will supplement the information gathered from B and C.

**Appendix 6: Daily Living Activity Record example**

| Time | 5/28 | 5/29 |
| --- | --- | --- |
| 0 |  | going to bed |
| 1 |  |  |
| 2 |  |  |
| 3 |  |  |
| 4 |  |  |
| 5 |  |  |
| 6 | Getting up |  |
| 7 | Washing clothes  Preparing for work |  |
| 8 | Going to office | Break fast |
| 9 | Desk work | picnic |
| 10 | Tea time | driving |
| 11 | meeting | Teatime |
| 12 | Desk work | Driving |
| 13 | Desk work | walking |
| 14 | Desk work | Lunch |
| 15 | Lunch | dancing |
| 16 |  | walking |
| 17 | Shopping to market on foot | driving |
| 18 | Taking bath | Friend house |
| 19 | cooking | Dinner together |
| 20 | dinner | chatting |
| 21 | Relaxing | chatting |
| 22 | Chatting with tablet | drive |
| 23 | sleeping | Preparing |
| Burning fat | 1.9g | 3.6g |
| Consumption | 2050Kcal | 2980Kcal |
| Distance | 2060m | 4070m |
| Steps | 6035steps | 10230steps |
| Run | 5min | 10min |
| walk | 35min | 120min |
| daily | 180min | 100min |
| Sleeping | 450min | 400min |

Fill up the date

During observation time, researcher fills up the memo.

Eg.) lunch time, walking, washing dishes, farming, grinding powder, what is he doing,

Non observation time, if participant can fill by himself, recommend filling up, but not possible, when researcher collect the colorism, researcher ask and fill up.

After filling up, scan data and pass them with explanation about consumption

Researcher will copy from Calorism memories.

Appendix 6: Budget estimate
