# Supplementary material for: A qualitative study on knowledge, perception, and practice related to non-communicable diseases in relation to happiness among rural and urban residents in Bhutan
Source: PLoS One. 2020 Jun 29;15(6):e0234257. doi: 10.1371/journal.pone.0234257 (PMC7323992; doi:10.1371/journal.pone.0234257)
Supplement: Attachment — Submitted filename: Response_to_Reviewers.docx [file pone.0234257.s002.docx]

Dear Editor and reviewers

I’m very appreciate of your review comments.

Reviewer #1: The manuscript should be revised linking how psychological well-being is part of happiness and why it matters for health through knowledge about NCDs. Also, the manuscript should refocus on health seeking behaviour, especially in context of NCDs.

⇒　To be clear on how psychological well-being is part of happiness and why it matters for health through knowledge about NCDs, I added a definition of GNH philosophy and my initial question in the introduction (Line 82 to 90).

Reviewer #2: The paper undoubtedly presents a very good initiative to consider happiness as an indicator of NCD prevalence in Bhutan albeit happiness is an abstract term and difficult to measure. The study undertook qualitative analysis while only IDI and observation study cannot define the state of happiness completely. A mixed methods approach could be excellent in this case to measure their property and land ownership and occupational status for which happiness state might vary. Also, there could be some potential confounders that needed to be considered in this study like, domestic violence, neighbourhood relations and most importantly subjective social status, i.e., occupational hierarchy that also evidently influence individuals' happiness and well-being. Also, there can be bias regarding the happiness related statements by the respondents. However, the authors did great by considering the traditional behaviour in this study on which mental satisfaction and happiness depend to a good extent.

Ultimately, this paper needs to discuss about other potential factors that have significant influence in determining happy or unhappy individuality. Furthermore, inclusion of quantitative analysis could provide more credible findings.

⇒We agree that a mixed-methods approach could be excellent to measure their property, land ownership, and occupational status for which happiness might vary. However, we thought that, if we conduct convergent design as mixed-method for happiness indicators, we could not explore their concept of health and happiness widely, nor their daily practice in life. We were then planning to conduct sequence design as a mixed-method and combine it with a survey of GNH or NCD in Bhutan. This study represents an initial phase of our research projects.

However, we did not mention the limitations and cofounders of happiness in this manuscript clearly, so we added an explanation of this in our discussion and limitations (Line 312 to 319, 361 to 362).
